# Supplementary figures and images for: Predicting errors in accident hotspots and investigating satiotemporal, weather, and behavioral factors using interpretable machine learning: An analysis of telematics big data
Source: PLoS One. 2025 Jul 8;20(7):e0326483. doi: 10.1371/journal.pone.0326483 (PMC12237018; doi:10.1371/journal.pone.0326483)

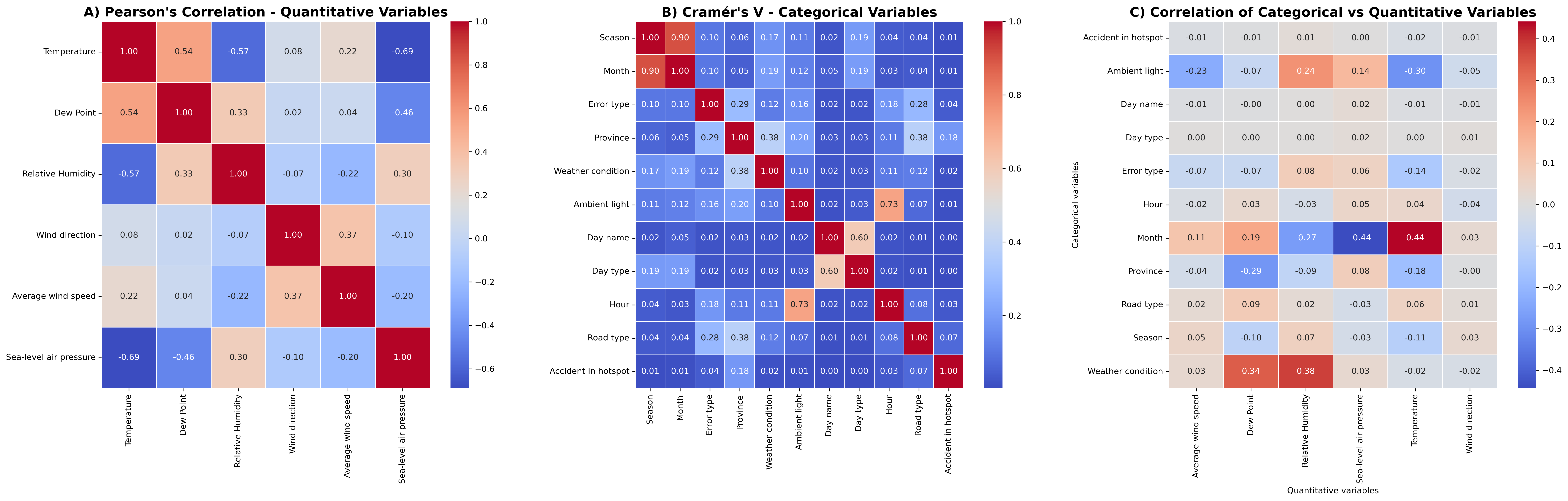

Supplement: S1 Fig — A) Pearson’s correlation for quantitative variables, B) Cramér’s V for categorical variables, C) Correlation of categorical vs quantitative variables. (PNG) [file pone.0326483.s001.png]

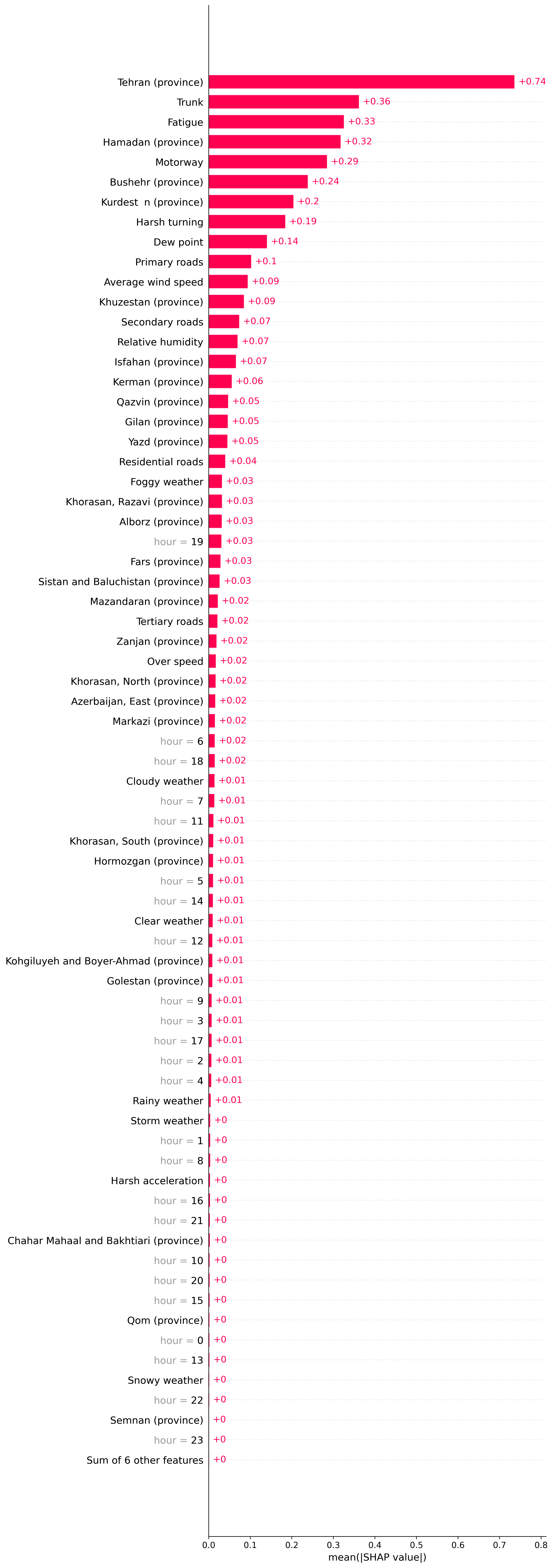

Supplement: S2 Fig — (PNG) [file pone.0326483.s002.png]

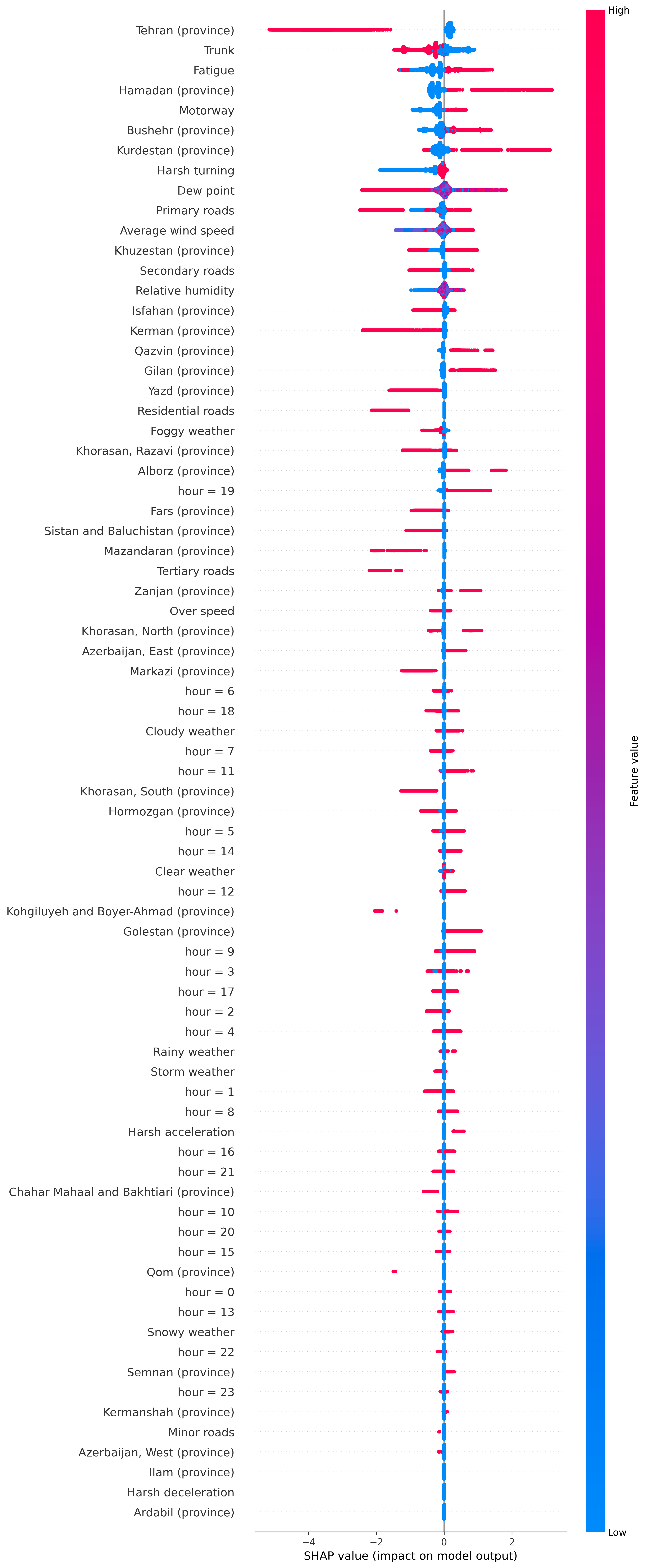

Supplement: S3 Fig — (PNG) [file pone.0326483.s003.png]
