# Supplementary material for: Predicting errors in accident hotspots and investigating satiotemporal, weather, and behavioral factors using interpretable machine learning: An analysis of telematics big data
Source: PLoS One. 2025 Jul 8;20(7):e0326483. doi: 10.1371/journal.pone.0326483 (PMC12237018; doi:10.1371/journal.pone.0326483)
Supplement: S1 Table — (DOCX) [file pone.0326483.s004.docx]

**S1 Table.** Distribution of accident hotspots in Iran.

| **Province** | **Number of hotspots** | **Percentage** |
| --- | --- | --- |
| **Fars** | 125 | 8.38 |
| **Khouzestan** | 120 | 8.04 |
| **North, Khorasan** | 96 | 6.43 |
| **Gilan** | 88 | 5.90 |
| **Kerman** | 85 | 5.70 |
| **Hamedan** | 70 | 4.69 |
| **Isfahan** | 68 | 4.56 |
| **Ghazvin** | 67 | 4.49 |
| **Ghom** | 66 | 4.42 |
| **East, Azarbayjan** | 63 | 4.22 |
| **Khorasan-Razavi** | 60 | 4.02 |
| **Tehran** | 55 | 3.69 |
| **Mazandaran** | 52 | 3.49 |
| **Kermanshah** | 49 | 3.28 |
| **Zanjan** | 46 | 3.08 |
| **Golestan** | 44 | 2.95 |
| **Lorestan** | 42 | 2.82 |
| **Yazd** | 38 | 2.55 |
| **West, Azarbayjan** | 34 | 2.28 |
| **Sistan and Baloucgestan** | 32 | 2.14 |
| **Alborz** | 31 | 2.08 |
| **Kordestan** | 28 | 1.88 |
| **Cheahr mahal and Bakhtiari** | 26 | 1.74 |
| **Ilam** | 22 | 1.47 |
| **Boushehr** | 20 | 1.34 |
| **Hormozgan** | 19 | 1.27 |
| **Semnan** | 18 | 1.21 |
| **South, KHorasan** | 16 | 1.07 |
| **Markazi** | 6 | 0.40 |
| **Ardabil** | 5 | 0.34 |
| **Kohgilouye and Boyer Ahmad** | 1 | 0.07 |
| **Total** | 1492 | 100.00 |
