# Supplementary material for: Predicting errors in accident hotspots and investigating satiotemporal, weather, and behavioral factors using interpretable machine learning: An analysis of telematics big data
Source: PLoS One. 2025 Jul 8;20(7):e0326483. doi: 10.1371/journal.pone.0326483 (PMC12237018; doi:10.1371/journal.pone.0326483)
Supplement: S2 Table — (DOCX) [file pone.0326483.s005.docx]

**Supplementary Table 2.** Descriptive summary of variables excluding weather-related variables in the primary dataset.

| **Variable** | **Error occurrence in accident hotspots (Number, percentage, 95% confidence interval)** | | **Total**  **(Number, percentage, 95% confidence interval)** |
| --- | --- | --- | --- |
|  | **No** | **Yes** |  |
| **Error type** |  | | |
| Harsh deceleration | 1411 (99.51%)(99.14-99.87) | 7 (0.49%)(0.13-0.86) | 1418 (0.09%)(0.08-0.09) |
| Harsh acceleration | 15403 (99.88%)(99.83-99.94) | 18 (0.12%)(0.06-0.17) | 15421 (0.97%)(0.96-0.99) |
| Harsh turning | 882120 (97.67%)(97.64-97.71) | 21004 (2.33%)(2.29-2.36) | 903124 (57.02%)(56.95-57.1) |
| Over speed | 194503 (98.97%)(98.93-99.02) | 2015 (1.03%)(0.98-1.07) | 196518 (12.41%)(12.36-12.46) |
| Fatigue | 460756 (98.59%)(98.56-98.63) | 6574 (1.41%)(1.37-1.44) | 467330 (29.51%)(29.44-29.58) |
| **Road Types** |  | | |
| trunk | 807402 (98.05%)(98.02-98.08) | 16049 (1.95%)(1.92-1.98) | 823451 (51.99%)(51.91-52.07) |
| motorway | 235650 (97.55%)(97.49-97.62) | 5910 (2.45%)(2.38-2.51) | 241560 (15.25%)(15.2-15.31) |
| primary | 326734 (98.19%)(98.15-98.24) | 6011 (1.81%)(1.76-1.85) | 332745 (21.01%)(20.95-21.07) |
| secondary | 113914 (98.76%)(98.69-98.82) | 1432 (1.24%)(1.18-1.31) | 115346 (7.28%)(7.24-7.32) |
| tertiary | 41600 (99.56%)(99.5-99.62) | 184 (0.44%)(0.38-0.5) | 41784 (2.64%)(2.61-2.66) |
| residential | 25479 (99.92%)(99.89-99.96) | 20 (0.08%)(0.04-0.11) | 25499 (1.61%)(1.59-1.63) |
| minor roads | 3414 (99.65%)(99.45-99.85) | 12 (0.35%)(0.15-0.55) | 3426 (0.22%)(0.21-0.22) |
| **Season** |  | | |
| Autumn | 409210 (98.18%)(98.14-98.22) | 7601 (1.82%)(1.78-1.86) | 416811 (26.32%)(26.25-26.39) |
| Spring | 296437 (98.1%)(98.06-98.15) | 5727 (1.9%)(1.85-1.94) | 302164 (19.08%)(19.02-19.14) |
| Summer | 405792 (98.06%)(98.01-98.1) | 8044 (1.94%)(1.9-1.99) | 413836 (26.13%)(26.06-26.2) |
| Winter | 442754 (98.17%)(98.13-98.21) | 8246 (1.83%)(1.79-1.87) | 451000 (28.48%)(28.41-28.55) |
| **Month** |  | | |
| January | 174309 (98.15%)(98.09-98.22) | 3280 (1.85%)(1.78-1.91) | 177589 (11.21%)(11.16-11.26) |
| February | 155370 (98.13%)(98.06-98.19) | 2967 (1.87%)(1.81-1.94) | 158337 (10.0%)(9.95-10.04) |
| March | 80220 (98.37%)(98.28-98.46) | 1329 (1.63%)(1.54-1.72) | 81549 (5.15%)(5.11-5.18) |
| April | 80316 (98.25%)(98.16-98.34) | 1430 (1.75%)(1.66-1.84) | 81746 (5.16%)(5.13-5.2) |
| May | 113599 (98.05%)(97.97-98.13) | 2254 (1.95%)(1.87-2.03) | 115853 (7.31%)(7.27-7.36) |
| June | 130300 (97.98%)(97.9-98.05) | 2691 (2.02%)(1.95-2.1) | 132991 (8.4%)(8.35-8.44) |
| July | 131044 (98.07%)(98.0-98.15) | 2576 (1.93%)(1.85-2.0) | 133620 (8.44%)(8.39-8.48) |
| August | 126511 (98.04%)(97.96-98.12) | 2529 (1.96%)(1.88-2.04) | 129040 (8.15%)(8.1-8.19) |
| September | 139083 (98.03%)(97.96-98.1) | 2792 (1.97%)(1.9-2.04) | 141875 (8.96%)(8.91-9.0) |
| October | 145414 (98.12%)(98.05-98.19) | 2790 (1.88%)(1.81-1.95) | 148204 (9.36%)(9.31-9.4) |
| November | 133100 (98.23%)(98.16-98.3) | 2395 (1.77%)(1.7-1.84) | 135495 (8.55%)(8.51-8.6) |
| December | 144927 (98.25%)(98.18-98.31) | 2585 (1.75%)(1.69-1.82) | 147512 (9.31%)(9.27-9.36) |
| **Ambient light situation** |  | | |
| daylight | 741175 (98.29%)(98.26-98.32) | 12869 (1.71%)(1.68-1.74) | 754044 (47.61%)(47.53-47.69) |
| nighttime | 657856 (97.93%)(97.9-97.97) | 13891 (2.07%)(2.03-2.1) | 671747 (42.41%)(42.34-42.49) |
| twilight | 155162 (98.19%)(98.13-98.26) | 2858 (1.81%)(1.74-1.87) | 158020 (9.98%)(9.93-10.02) |
| **Day name** |  | | |
| Friday | 205304 (98.06%)(98.0-98.12) | 4059 (1.94%)(1.88-2.0) | 209363 (13.22%)(13.17-13.27) |
| Monday | 220217 (98.08%)(98.02-98.14) | 4309 (1.92%)(1.86-1.98) | 224526 (14.18%)(14.12-14.23) |
| Saturday | 227097 (98.22%)(98.17-98.27) | 4116 (1.78%)(1.73-1.83) | 231213 (14.6%)(14.54-14.65) |
| Sunday | 232270 (98.17%)(98.12-98.23) | 4327 (1.83%)(1.77-1.88) | 236597 (14.94%)(14.88-14.99) |
| Thursday | 218407 (98.13%)(98.07-98.18) | 4170 (1.87%)(1.82-1.93) | 222577 (14.05%)(14.0-14.11) |
| Tuesday | 221658 (98.12%)(98.07-98.18) | 4236 (1.88%)(1.82-1.93) | 225894 (14.26%)(14.21-14.32) |
| Wednesday | 229240 (98.12%)(98.06-98.17) | 4401 (1.88%)(1.83-1.94) | 233641 (14.75%)(14.7-14.81) |
| **Day type** |  | | |
| Friday | 175018 (98.08%)(98.01-98.14) | 3433 (1.92%)(1.86-1.99) | 178451 (11.27%)(11.22-11.32) |
| Holiday | 23513 (98.03%)(97.86-98.21) | 472 (1.97%)(1.79-2.14) | 23985 (1.51%)(1.5-1.53) |
| Long holidays | 90782 (98.14%)(98.05-98.23) | 1722 (1.86%)(1.77-1.95) | 92504 (5.84%)(5.8-5.88) |
| Pre long holidays | 34425 (98.07%)(97.93-98.22) | 677 (1.93%)(1.78-2.07) | 35102 (2.22%)(2.19-2.24) |
| Thursday | 186787 (98.13%)(98.07-98.19) | 3564 (1.87%)(1.81-1.93) | 190351 (12.02%)(11.97-12.07) |
| Workdays | 1043668 (98.14%)(98.12-98.17) | 19750 (1.86%)(1.83-1.88) | 1063418 (67.14%)(67.07-67.22) |
| **Hour** |  |  |  |
| 0 | 54559 (97.52%)(97.39-97.65) | 1388 (2.48%)(2.35-2.61) | 55947 (3.53%)(3.5-3.56) |
| 1 | 63802 (97.74%)(97.63-97.85) | 1475 (2.26%)(2.15-2.37) | 65277 (4.12%)(4.09-4.15) |
| 2 | 78362 (97.85%)(97.75-97.95) | 1722 (2.15%)(2.05-2.25) | 80084 (5.06%)(5.02-5.09) |
| 3 | 86234 (97.71%)(97.61-97.81) | 2022 (2.29%)(2.19-2.39) | 88256 (5.57%)(5.54-5.61) |
| 4 | 79105 (97.8%)(97.7-97.9) | 1779 (2.2%)(2.1-2.3) | 80884 (5.11%)(5.07-5.14) |
| 5 | 77886 (98.13%)(98.03-98.22) | 1488 (1.87%)(1.78-1.97) | 79374 (5.01%)(4.98-5.05) |
| 6 | 88279 (98.34%)(98.26-98.43) | 1488 (1.66%)(1.57-1.74) | 89767 (5.67%)(5.63-5.7) |
| 7 | 77382 (98.67%)(98.59-98.75) | 1045 (1.33%)(1.25-1.41) | 78427 (4.95%)(4.92-4.99) |
| 8 | 58098 (98.8%)(98.71-98.89) | 707 (1.2%)(1.11-1.29) | 58805 (3.71%)(3.68-3.74) |
| 9 | 48698 (98.46%)(98.35-98.57) | 763 (1.54%)(1.43-1.65) | 49461 (3.12%)(3.1-3.15) |
| 10 | 46618 (98.54%)(98.44-98.65) | 689 (1.46%)(1.35-1.56) | 47307 (2.99%)(2.96-3.01) |
| 11 | 48098 (98.44%)(98.33-98.55) | 760 (1.56%)(1.45-1.67) | 48858 (3.08%)(3.06-3.11) |
| 12 | 49775 (98.43%)(98.32-98.54) | 795 (1.57%)(1.46-1.68) | 50570 (3.19%)(3.17-3.22) |
| 13 | 55593 (98.27%)(98.17-98.38) | 977 (1.73%)(1.62-1.83) | 56570 (3.57%)(3.54-3.6) |
| 14 | 59682 (97.83%)(97.71-97.94) | 1325 (2.17%)(2.06-2.29) | 61007 (3.85%)(3.82-3.88) |
| 15 | 57060 (97.81%)(97.69-97.92) | 1280 (2.19%)(2.08-2.31) | 58340 (3.68%)(3.65-3.71) |
| 16 | 69412 (98.15%)(98.06-98.25) | 1305 (1.85%)(1.75-1.94) | 70717 (4.46%)(4.43-4.5) |
| 17 | 73503 (98.1%)(98.01-98.2) | 1420 (1.9%)(1.8-1.99) | 74923 (4.73%)(4.7-4.76) |
| 18 | 79693 (98.27%)(98.18-98.36) | 1400 (1.73%)(1.64-1.82) | 81093 (5.12%)(5.09-5.15) |
| 19 | 81109 (98.19%)(98.1-98.28) | 1495 (1.81%)(1.72-1.9) | 82604 (5.22%)(5.18-5.25) |
| 20 | 67335 (98.18%)(98.08-98.28) | 1245 (1.82%)(1.72-1.92) | 68580 (4.33%)(4.3-4.36) |
| 21 | 56581 (97.89%)(97.77-98.0) | 1222 (2.11%)(2.0-2.23) | 57803 (3.65%)(3.62-3.68) |
| 22 | 48594 (98.26%)(98.15-98.38) | 860 (1.74%)(1.62-1.85) | 49454 (3.12%)(3.1-3.15) |
| 23 | 48735 (98.05%)(97.93-98.17) | 968 (1.95%)(1.83-2.07) | 49703 (3.14%)(3.11-3.17) |
| **Province** |  |  |  |
| Alborz | 48770 (98.01%)(97.89-98.14) | 989 (1.99%)(1.86-2.11) | 49759 (3.14%)(3.11-3.17) |
| Ardabil | 24379 (99.98%)(99.96-100.0) | 5 (0.02%)(0.0-0.04) | 24384 (1.54%)(1.52-1.56) |
| Azerbaijan, East | 54550 (98.45%)(98.35-98.55) | 858 (1.55%)(1.45-1.65) | 55408 (3.5%)(3.47-3.53) |
| Azerbaijan, West | 7336 (99.66%)(99.53-99.79) | 25 (0.34%)(0.21-0.47) | 7361 (0.46%)(0.45-0.48) |
| Bushehr | 189520 (98.58%)(98.53-98.64) | 2722 (1.42%)(1.36-1.47) | 192242 (12.14%)(12.09-12.19) |
| Chahar Mahaal and Bakhtiari | 14824 (99.25%)(99.11-99.39) | 112 (0.75%)(0.61-0.89) | 14936 (0.94%)(0.93-0.96) |
| Fars | 98466 (95.94%)(95.81-96.06) | 4172 (4.06%)(3.94-4.19) | 102638 (6.48%)(6.44-6.52) |
| Gilan | 38387 (98.56%)(98.44-98.68) | 560 (1.44%)(1.32-1.56) | 38947 (2.46%)(2.43-2.48) |
| Golestan | 11911 (89.48%)(88.95-90.0) | 1401 (10.52%)(10.0-11.05) | 13312 (0.84%)(0.83-0.85) |
| Hamadan | 34070 (93.39%)(93.14-93.65) | 2411 (6.61%)(6.35-6.86) | 36481 (2.3%)(2.28-2.33) |
| Hormozgan | 17683 (99.8%)(99.73-99.86) | 36 (0.2%)(0.14-0.27) | 17719 (1.12%)(1.1-1.14) |
| Ilam | 23190 (96.67%)(96.45-96.9) | 798 (3.33%)(3.1-3.55) | 23988 (1.51%)(1.5-1.53) |
| Isfahan | 206316 (99.49%)(99.46-99.52) | 1059 (0.51%)(0.48-0.54) | 207375 (13.09%)(13.04-13.15) |
| Kerman | 45796 (99.79%)(99.74-99.83) | 98 (0.21%)(0.17-0.26) | 45894 (2.9%)(2.87-2.92) |
| Kermanshah | 12579 (98.63%)(98.43-98.83) | 175 (1.37%)(1.17-1.57) | 12754 (0.81%)(0.79-0.82) |
| Khorasan, North | 19173 (98.7%)(98.54-98.86) | 252 (1.3%)(1.14-1.46) | 19425 (1.23%)(1.21-1.24) |
| Khorasan, Razavi | 38295 (99.34%)(99.25-99.42) | 256 (0.66%)(0.58-0.75) | 38551 (2.43%)(2.41-2.46) |
| Khorasan, South | 15165 (99.73%)(99.65-99.81) | 41 (0.27%)(0.19-0.35) | 15206 (0.96%)(0.94-0.98) |
| Khuzestan | 70383 (94.34%)(94.17-94.5) | 4226 (5.66%)(5.5-5.83) | 74609 (4.71%)(4.68-4.74) |
| Kohgiluyeh and Boyer-Ahmad | 11664 (100.0%)(100.0-100.0) | 0 (0.0%)(0.0-0.0) | 11664 (0.74%)(0.72-0.75) |
| Kurdistan | 44846 (93.54%)(93.32-93.76) | 3097 (6.46%)(6.24-6.68) | 47943 (3.03%)(3.0-3.05) |
| Lorestan | 35153 (98.83%)(98.72-98.94) | 416 (1.17%)(1.06-1.28) | 35569 (2.25%)(2.22-2.27) |
| Markazi | 30323 (99.76%)(99.7-99.81) | 74 (0.24%)(0.19-0.3) | 30397 (1.92%)(1.9-1.94) |
| Mazandaran | 43148 (99.1%)(99.01-99.18) | 394 (0.9%)(0.82-0.99) | 43542 (2.75%)(2.72-2.77) |
| Qazvin | 25718 (96.3%)(96.08-96.53) | 987 (3.7%)(3.47-3.92) | 26705 (1.69%)(1.67-1.71) |
| Qom | 53856 (96.14%)(95.98-96.29) | 2165 (3.86%)(3.71-4.02) | 56021 (3.54%)(3.51-3.57) |
| Semnan | 18286 (99.46%)(99.35-99.56) | 100 (0.54%)(0.44-0.65) | 18386 (1.16%)(1.14-1.18) |
| Sistan and Baluchistan | 33773 (99.85%)(99.81-99.89) | 51 (0.15%)(0.11-0.19) | 33824 (2.14%)(2.11-2.16) |
| Tehran | 204730 (99.67%)(99.65-99.7) | 673 (0.33%)(0.3-0.35) | 205403 (12.97%)(12.92-13.02) |
| Yazd | 36952 (99.72%)(99.66-99.77) | 105 (0.28%)(0.23-0.34) | 37057 (2.34%)(2.32-2.36) |
| Zanjan | 44951 (97.06%)(96.91-97.22) | 1360 (2.94%)(2.78-3.09) | 46311 (2.92%)(2.9-2.95) |
| **Total** | **1554193 (98.13%)(98.11-98.15)** | **29618 (1.87%)(1.85-1.89)** | **1583811** |
