# Supplementary material for: Predicting errors in accident hotspots and investigating satiotemporal, weather, and behavioral factors using interpretable machine learning: An analysis of telematics big data
Source: PLoS One. 2025 Jul 8;20(7):e0326483. doi: 10.1371/journal.pone.0326483 (PMC12237018; doi:10.1371/journal.pone.0326483)
Supplement: S3 Table — (DOCX) [file pone.0326483.s006.docx]

**S3 Table.** Descriptive summary of variables in the dataset after prediction of weather condition and without any missing values.

| **Categorical variables** | | | |
| --- | --- | --- | --- |
| **Variable** | **Error occurrence in accident hotspots (Number, percentage, 95% confidence interval)** | | **Total**  **(Number, percentage, 95% confidence interval)** |
|  | **No** | **Yes** |  |
| **Error type** |  | | |
| Harsh deceleration | 257 (100.0%)(100.0-100.0) | 0 (0.0%)(0.0-0.0) | 257 (0.04%)(0.04-0.05) |
| Harsh acceleration | 6457 (99.86%)(99.77-99.95) | 9 (0.14%)(0.05-0.23) | 6466 (1.04%)(1.02-1.07) |
| Harsh turning | 360039 (98.04%)(97.99-98.08) | 7215 (1.96%)(1.92-2.01) | 367254 (59.24%)(59.11-59.36) |
| Over speed | 75411 (99.32%)(99.26-99.38) | 517 (0.68%)(0.62-0.74) | 75928 (12.25%)(12.17-12.33) |
| Fatigue | 167991 (98.77%)(98.72-98.82) | 2092 (1.23%)(1.18-1.28) | 170083 (27.43%)(27.32-27.54) |
| **Road Types** |  | | |
| trunk | 339298 (98.58%)(98.54-98.62) | 4894 (1.42%)(1.38-1.46) | 344192 (55.52%)(55.39-55.64) |
| motorway | 84386 (97.84%)(97.75-97.94) | 1861 (2.16%)(2.06-2.25) | 86247 (13.91%)(13.82-14.0) |
| primary | 89784 (96.84%)(96.73-96.96) | 2925 (3.16%)(3.04-3.27) | 92709 (14.95%)(14.86-15.04) |
| secondary | 75003 (99.8%)(99.77-99.83) | 150 (0.2%)(0.17-0.23) | 75153 (12.12%)(12.04-12.2) |
| tertiary | 7607 (99.99%)(99.96-100.0) | 1 (0.01%)(0.0-0.04) | 7608 (1.23%)(1.2-1.25) |
| residential | 1300 (100.0%)(100.0-100.0) | 0 (0.0%)(0.0-0.0) | 1300 (0.21%)(0.2-0.22) |
| minor roads | 12777 (99.98%)(99.96-100.0) | 2 (0.02%)(0.0-0.04) | 12779 (2.06%)(2.03-2.1) |
| **Season** |  | | |
| Autumn | 157033 (98.38%)(98.32-98.45) | 2579 (1.62%)(1.55-1.68) | 159612 (25.74%)(25.64-25.85) |
| Spring | 123078 (98.43%)(98.36-98.5) | 1964 (1.57%)(1.5-1.64) | 125042 (20.17%)(20.07-20.27) |
| Summer | 163410 (98.31%)(98.25-98.37) | 2812 (1.69%)(1.63-1.75) | 166222 (26.81%)(26.7-26.92) |
| Winter | 166634 (98.53%)(98.48-98.59) | 2478 (1.47%)(1.41-1.52) | 169112 (27.28%)(27.17-27.39) |
| **Month** |  | | |
| January | 60180 (98.54%)(98.44-98.63) | 893 (1.46%)(1.37-1.56) | 61073 (9.85%)(9.78-9.92) |
| February | 60010 (98.4%)(98.3-98.5) | 977 (1.6%)(1.5-1.7) | 60987 (9.84%)(9.76-9.91) |
| March | 35545 (98.82%)(98.71-98.93) | 424 (1.18%)(1.07-1.29) | 35969 (5.8%)(5.74-5.86) |
| April | 34560 (98.58%)(98.45-98.7) | 499 (1.42%)(1.3-1.55) | 35059 (5.65%)(5.6-5.71) |
| May | 46491 (98.32%)(98.2-98.43) | 796 (1.68%)(1.57-1.8) | 47287 (7.63%)(7.56-7.69) |
| June | 51075 (98.27%)(98.16-98.38) | 900 (1.73%)(1.62-1.84) | 51975 (8.38%)(8.31-8.45) |
| July | 52681 (98.33%)(98.22-98.44) | 893 (1.67%)(1.56-1.78) | 53574 (8.64%)(8.57-8.71) |
| August | 51840 (98.23%)(98.12-98.35) | 932 (1.77%)(1.65-1.88) | 52772 (8.51%)(8.44-8.58) |
| September | 55502 (98.34%)(98.24-98.45) | 936 (1.66%)(1.55-1.76) | 56438 (9.1%)(9.03-9.17) |
| October | 55686 (98.27%)(98.16-98.38) | 981 (1.73%)(1.62-1.84) | 56667 (9.14%)(9.07-9.21) |
| November | 50675 (98.45%)(98.34-98.55) | 799 (1.55%)(1.45-1.66) | 51474 (8.3%)(8.23-8.37) |
| December | 55910 (98.58%)(98.49-98.68) | 803 (1.42%)(1.32-1.51) | 56713 (9.15%)(9.08-9.22) |
| **Ambient light situation** |  | | |
| daylight | 336068 (98.54%)(98.5-98.58) | 4974 (1.46%)(1.42-1.5) | 341042 (55.01%)(54.88-55.13) |
| nighttime | 210165 (98.21%)(98.15-98.26) | 3839 (1.79%)(1.74-1.85) | 214004 (34.52%)(34.4-34.64) |
| twilight | 63922 (98.43%)(98.33-98.52) | 1020 (1.57%)(1.48-1.67) | 64942 (10.47%)(10.4-10.55) |
| **Day name** |  | | |
| Friday | 78142 (98.39%)(98.3-98.47) | 1282 (1.61%)(1.53-1.7) | 79424 (12.81%)(12.73-12.89) |
| Monday | 86078 (98.35%)(98.27-98.44) | 1441 (1.65%)(1.56-1.73) | 87519 (14.12%)(14.03-14.2) |
| Saturday | 89393 (98.49%)(98.41-98.56) | 1375 (1.51%)(1.44-1.59) | 90768 (14.64%)(14.55-14.73) |
| Sunday | 92371 (98.45%)(98.37-98.53) | 1453 (1.55%)(1.47-1.63) | 93824 (15.13%)(15.04-15.22) |
| Thursday | 84893 (98.42%)(98.34-98.5) | 1364 (1.58%)(1.5-1.66) | 86257 (13.91%)(13.83-14.0) |
| Tuesday | 89139 (98.39%)(98.31-98.47) | 1458 (1.61%)(1.53-1.69) | 90597 (14.61%)(14.52-14.7) |
| Wednesday | 90139 (98.41%)(98.32-98.49) | 1460 (1.59%)(1.51-1.68) | 91599 (14.77%)(14.69-14.86) |
| **Day type** |  | | |
| Friday | 65949 (98.4%)(98.31-98.5) | 1072 (1.6%)(1.5-1.69) | 67021 (10.81%)(10.73-10.89) |
| Holiday | 9122 (98.35%)(98.09-98.61) | 153 (1.65%)(1.39-1.91) | 9275 (1.5%)(1.47-1.53) |
| Long holidays | 38358 (98.5%)(98.38-98.62) | 585 (1.5%)(1.38-1.62) | 38943 (6.28%)(6.22-6.34) |
| Pre long holidays | 14168 (98.33%)(98.12-98.54) | 241 (1.67%)(1.46-1.88) | 14409 (2.32%)(2.29-2.36) |
| Thursday | 71955 (98.42%)(98.33-98.51) | 1157 (1.58%)(1.49-1.67) | 73112 (11.79%)(11.71-11.87) |
| Workdays | 410603 (98.41%)(98.37-98.45) | 6625 (1.59%)(1.55-1.63) | 417228 (67.3%)(67.18-67.41) |
| **Hour** |  | | |
| 0 | 16664 (97.37%)(97.13-97.61) | 450 (2.63%)(2.39-2.87) | 17114 (2.76%)(2.72-2.8) |
| 1 | 15806 (97.99%)(97.77-98.21) | 324 (2.01%)(1.79-2.23) | 16130 (2.6%)(2.56-2.64) |
| 2 | 21664 (98.33%)(98.16-98.49) | 369 (1.67%)(1.51-1.84) | 22033 (3.55%)(3.51-3.6) |
| 3 | 22749 (97.68%)(97.48-97.87) | 541 (2.32%)(2.13-2.52) | 23290 (3.76%)(3.71-3.8) |
| 4 | 24045 (97.98%)(97.8-98.15) | 496 (2.02%)(1.85-2.2) | 24541 (3.96%)(3.91-4.01) |
| 5 | 31290 (98.78%)(98.66-98.91) | 385 (1.22%)(1.09-1.34) | 31675 (5.11%)(5.05-5.16) |
| 6 | 47036 (98.94%)(98.84-99.03) | 506 (1.06%)(0.97-1.16) | 47542 (7.67%)(7.6-7.73) |
| 7 | 41514 (98.96%)(98.87-99.06) | 435 (1.04%)(0.94-1.13) | 41949 (6.77%)(6.7-6.83) |
| 8 | 29721 (98.88%)(98.76-98.99) | 338 (1.12%)(1.01-1.24) | 30059 (4.85%)(4.79-4.9) |
| 9 | 22001 (98.6%)(98.44-98.75) | 313 (1.4%)(1.25-1.56) | 22314 (3.6%)(3.55-3.65) |
| 10 | 18542 (98.53%)(98.36-98.7) | 277 (1.47%)(1.3-1.64) | 18819 (3.04%)(2.99-3.08) |
| 11 | 19931 (98.29%)(98.12-98.47) | 346 (1.71%)(1.53-1.88) | 20277 (3.27%)(3.23-3.31) |
| 12 | 20157 (98.56%)(98.39-98.72) | 295 (1.44%)(1.28-1.61) | 20452 (3.3%)(3.25-3.34) |
| 13 | 23782 (98.34%)(98.18-98.5) | 402 (1.66%)(1.5-1.82) | 24184 (3.9%)(3.85-3.95) |
| 14 | 27065 (98.29%)(98.14-98.45) | 470 (1.71%)(1.55-1.86) | 27535 (4.44%)(4.39-4.49) |
| 15 | 22904 (98.41%)(98.25-98.57) | 371 (1.59%)(1.43-1.75) | 23275 (3.75%)(3.71-3.8) |
| 16 | 27425 (98.33%)(98.18-98.48) | 465 (1.67%)(1.52-1.82) | 27890 (4.5%)(4.45-4.55) |
| 17 | 32435 (98.14%)(97.99-98.28) | 615 (1.86%)(1.72-2.01) | 33050 (5.33%)(5.27-5.39) |
| 18 | 34588 (98.43%)(98.3-98.56) | 552 (1.57%)(1.44-1.7) | 35140 (5.67%)(5.61-5.73) |
| 19 | 34583 (98.27%)(98.13-98.4) | 610 (1.73%)(1.6-1.87) | 35193 (5.68%)(5.62-5.73) |
| 20 | 24815 (98.64%)(98.5-98.79) | 341 (1.36%)(1.21-1.5) | 25156 (4.06%)(4.01-4.11) |
| 21 | 18211 (98.6%)(98.43-98.77) | 258 (1.4%)(1.23-1.57) | 18469 (2.98%)(2.94-3.02) |
| 22 | 16172 (98.27%)(98.07-98.47) | 285 (1.73%)(1.53-1.93) | 16457 (2.65%)(2.61-2.69) |
| 23 | 17055 (97.77%)(97.55-97.99) | 389 (2.23%)(2.01-2.45) | 17444 (2.81%)(2.77-2.85) |
| **Province** |  | | |
| Alborz | 4995 (94.78%)(94.18-95.38) | 275 (5.22%)(4.62-5.82) | 5270 (0.85%)(0.83-0.87) |
| Ardabil | 137 (100.0%)(100.0-100.0) | 0 (0.0%)(0.0-0.0) | 137 (0.02%)(0.02-0.03) |
| Azerbaijan, East | 12419 (98.38%)(98.16-98.6) | 205 (1.62%)(1.4-1.84) | 12624 (2.04%)(2.0-2.07) |
| Azerbaijan, West | 871 (99.32%)(98.77-99.86) | 6 (0.68%)(0.14-1.23) | 877 (0.14%)(0.13-0.15) |
| Bushehr | 144214 (98.26%)(98.19-98.33) | 2551 (1.74%)(1.67-1.81) | 146765 (23.67%)(23.57-23.78) |
| Chahar Mahaal and Bakhtiari | 3117 (99.87%)(99.75-100.0) | 4 (0.13%)(0.0-0.25) | 3121 (0.5%)(0.49-0.52) |
| Fars | 22004 (99.73%)(99.66-99.8) | 59 (0.27%)(0.2-0.34) | 22063 (3.56%)(3.51-3.6) |
| Gilan | 12212 (98.21%)(97.97-98.44) | 223 (1.79%)(1.56-2.03) | 12435 (2.01%)(1.97-2.04) |
| Golestan | 2975 (97.35%)(96.78-97.92) | 81 (2.65%)(2.08-3.22) | 3056 (0.49%)(0.48-0.51) |
| Hamadan | 18694 (89.84%)(89.43-90.25) | 2114 (10.16%)(9.75-10.57) | 20808 (3.36%)(3.31-3.4) |
| Hormozgan | 11629 (99.84%)(99.76-99.91) | 19 (0.16%)(0.09-0.24) | 11648 (1.88%)(1.84-1.91) |
| Ilam | 2 (100.0%)(100.0-100.0) | 0 (0.0%)(0.0-0.0) | 2 (0.0%)(0.0-0.0) |
| Isfahan | 90987 (99.35%)(99.3-99.4) | 596 (0.65%)(0.6-0.7) | 91583 (14.77%)(14.68-14.86) |
| Kerman | 14991 (99.94%)(99.9-99.98) | 9 (0.06%)(0.02-0.1) | 15000 (2.42%)(2.38-2.46) |
| Kermanshah | 5292 (99.44%)(99.24-99.64) | 30 (0.56%)(0.36-0.76) | 5322 (0.86%)(0.84-0.88) |
| Khorasan, North | 12626 (99.21%)(99.06-99.37) | 100 (0.79%)(0.63-0.94) | 12726 (2.05%)(2.02-2.09) |
| Khorasan, Razavi | 16073 (99.73%)(99.65-99.81) | 43 (0.27%)(0.19-0.35) | 16116 (2.6%)(2.56-2.64) |
| Khorasan, South | 5116 (99.82%)(99.71-99.94) | 9 (0.18%)(0.06-0.29) | 5125 (0.83%)(0.8-0.85) |
| Khuzestan | 24181 (97.88%)(97.7-98.05) | 525 (2.12%)(1.95-2.3) | 24706 (3.98%)(3.94-4.03) |
| Kohgiluyeh and Boyer-Ahmad | 2211 (100.0%)(100.0-100.0) | 0 (0.0%)(0.0-0.0) | 2211 (0.36%)(0.34-0.37) |
| Kordestan | 15569 (90.77%)(90.34-91.2) | 1583 (9.23%)(8.8-9.66) | 17152 (2.77%)(2.73-2.81) |
| Markazi | 6491 (99.74%)(99.61-99.86) | 17 (0.26%)(0.14-0.39) | 6508 (1.05%)(1.02-1.08) |
| Mazandaran | 5336 (99.96%)(99.91-100.0) | 2 (0.04%)(0.0-0.09) | 5338 (0.86%)(0.84-0.88) |
| Qazvin | 16781 (96.08%)(95.79-96.37) | 685 (3.92%)(3.63-4.21) | 17466 (2.82%)(2.78-2.86) |
| Qom | 550 (100.0%)(100.0-100.0) | 0 (0.0%)(0.0-0.0) | 550 (0.09%)(0.08-0.1) |
| Semnan | 3416 (99.07%)(98.75-99.39) | 32 (0.93%)(0.61-1.25) | 3448 (0.56%)(0.54-0.57) |
| Sistan and Baluchistan | 14695 (99.78%)(99.71-99.86) | 32 (0.22%)(0.14-0.29) | 14727 (2.38%)(2.34-2.41) |
| Tehran | 100365 (99.97%)(99.96-99.98) | 31 (0.03%)(0.02-0.04) | 100396 (16.19%)(16.1-16.28) |
| Yazd | 18708 (99.86%)(99.81-99.91) | 26 (0.14%)(0.09-0.19) | 18734 (3.02%)(2.98-3.06) |
| Zanjan | 23498 (97.61%)(97.41-97.8) | 576 (2.39%)(2.2-2.59) | 24074 (3.88%)(3.83-3.93) |
| Alborz | 4995 (94.78%)(94.18-95.38) | 275 (5.22%)(4.62-5.82) | 5270 (0.85%)(0.83-0.87) |
| **Weather condition** |  | | |
| Clear | 318693 (98.29%)(98.25-98.34) | 5533 (1.71%)(1.66-1.75) | 324226 (52.3%)(52.17-52.42) |
| Cloudy | 36291 (98.21%)(98.07-98.34) | 663 (1.79%)(1.66-1.93) | 36954 (5.96%)(5.9-6.02) |
| Foggy | 202004 (98.61%)(98.56-98.66) | 2843 (1.39%)(1.34-1.44) | 204847 (33.04%)(32.92-33.16) |
| Rainy | 32872 (98.5%)(98.37-98.63) | 500 (1.5%)(1.37-1.63) | 33372 (5.38%)(5.33-5.44) |
| Snowy | 6594 (97.4%)(97.02-97.78) | 176 (2.6%)(2.22-2.98) | 6770 (1.09%)(1.07-1.12) |
| Storm | 13701 (99.15%)(98.99-99.3) | 118 (0.85%)(0.7-1.01) | 13819 (2.23%)(2.19-2.27) |
| **Total** | **610155 (98.41%)(98.38-98.45)** | **9833 (1.59%)(1.55-1.62)** | **619988** |
| **Quantitative variables** | | | |
| **Variable (unit)** | **Error occurrence in accident hotspots (Mean ± standard deviation)** | | **total** |
|  | **No** | **Yes** |  |
| Temperature (°C) | 19.82 ± 11.73 | 18.07 ± 12.00 | 19.79 ± 11.74 |
| Dew point (°C) | 4.60 ± 9.62 | 4.17 ± 8.11 | 4.59 ± 9.59 |
| relative humidity (%) | 45.03 ± 25.32 | 48.05 ± 25.59 | 45.08 ± 25.33 |
| wind direction (degrees) | 171.72 ± 113.51 | 162.54 ± 111.96 | 171.57 ± 113.50 |
| average wind speed (kilometers per hour) | 10.18 ± 7.54 | 9.41 ± 7.45 | 10.17 ± 7.54 |
| sea-level air pressure (hPa) | 1013.85 ± 8.50 | 1013.98 ± 7.97 | 1013.86 ± 8.49 |
| * °C: Celsius, hPa: hectopascal | | | |
