# Supplementary material for: Predicting errors in accident hotspots and investigating satiotemporal, weather, and behavioral factors using interpretable machine learning: An analysis of telematics big data
Source: PLoS One. 2025 Jul 8;20(7):e0326483. doi: 10.1371/journal.pone.0326483 (PMC12237018; doi:10.1371/journal.pone.0326483)
Supplement: S4 Table — (DOCX) [file pone.0326483.s007.docx]

**Supplementary Table 4.** Models hyperparameters tuning.

| **Model name** | **Hyperparameters tuning** | **Hyperparameters of best performing model** |
| --- | --- | --- |
| Logistic regression | solver = ['newton-cg', 'saga']  penalty = ['none', 'l1', 'l2', '‘elasticnet’']  C = [100, 10, 1.0, 0.1, 0.01] | best_params = {  'solver': 'newton-cg',  'penalty': 'l2',  'C': 10  } |
| K-nearest neighbors (KNN) | n_neighbors = [3, 5, 7]  weights = ['uniform']  metric = ['euclidean'] | best_params = {  'n_neighbors': 7,  'weights':'uniform',  'metric':'euclidean'  } |
| random forest (RF) | max_depth = [10, 50, 100]  n_estimators = [200, 600, 1000, 1400] | best_params = {  'max_depth': 50,  'n_estimators': 1400,  } |
| Extreme Gradient Boosting (XGBoost) | max_depth = [5, 10, 20]  n_estimators = [10, 100, 1000]  learning_rate = [0.01, 0.1] | best_params = {  'max_depth': 10,  'n_estimators': 1000,  'learning_rate': 0.01  } |
| Naïve Bayes | alpha = [1.0, 0.1, 0.01, 0.001] | best_params = {  'alpha': 0.001  } |
| Support vector machine (SVM) | C = [ 0.01, 1, 100]  penalty= ['l2'] | best_params = {  'C': 100  } |
| * For other hyperparameters, the default values provided by the library were used in the model tuning process. | | |
