# Supplementary material for: Predicting errors in accident hotspots and investigating satiotemporal, weather, and behavioral factors using interpretable machine learning: An analysis of telematics big data
Source: PLoS One. 2025 Jul 8;20(7):e0326483. doi: 10.1371/journal.pone.0326483 (PMC12237018; doi:10.1371/journal.pone.0326483)
Supplement: S6 Table — (DOCX) [file pone.0326483.s009.docx]

**S6 Table.** Evaluation of machine learning models for the prediction of error occurrence in accident hotspots.

| **Model name** | **Recall (%)** | **Precision (%)** | **F1-score (%)** | **Weighted F1-score (%)** | **Accuracy (%)** | **Balanced accuracy (%)** | **AUC (%) (95% UI)** |
| --- | --- | --- | --- | --- | --- | --- | --- |
| Logistic regression | 83.37 | 5.34 | 10.04 | 84.94 | 76.02 | 79.63 | 85.73 (85.15-86.28) |
| K-nearest neighbors (KNN) | 88.22 | 5.66 | 10.63 | 84.83 | 76.33 | 82.19 | 90.09 (89.60 – 90.58) |
| Random forest (RF) | 86.73 | 7.22 | 13.33 | 88.66 | 81.89 | 84.27 | 91.14 (90.72 - 91.55) |
| Extreme Gradient Boosting (XGBoost) | 88.44 | 7.05 | 13.06 | 88.16 | 81.09 | 84.70 | 91.70 (91.33 – 92.09) |
| Naïve Bayes | 84.14 | 4.47 | 8.50 | 81.51 | 70.90 | 77.41 | 84.77 (84.09 - 85.40) |
| Support vector machine (SVM) | 83.30 | 5.32 | 10.01 | 84.90 | 75.95 | 79.57 | 82.03 (81.37 - 82.66) |
