# Supplementary material for: Predicting errors in accident hotspots and investigating satiotemporal, weather, and behavioral factors using interpretable machine learning: An analysis of telematics big data
Source: PLoS One. 2025 Jul 8;20(7):e0326483. doi: 10.1371/journal.pone.0326483 (PMC12237018; doi:10.1371/journal.pone.0326483)
Supplement: Attachment — Submitted filename: Response to Editor.pdf [file pone.0326483.s013.pdf]

Response to Editor:

Editor:

The overall quality of the manuscript is good, but it is important to address a few comments before publication, as this journal maintains a high standard quality. This is also vital for the reputation of all experts involved in this manuscript, including the reviewers, the editor, and the esteemed authors.

Please recheck all English copyediting and grammatical issues. For example, in the third sentence of your introduction, change:

"93% of road traffic deaths occurred in low- and middle-income countries (LMIC), despite they have only 60% of vehicles [1]"

to

"About 93% of road traffic deaths occurred in low- and middle-income countries (LMIC), despite having only 60% of vehicles [1]."

Do not start sentences with numerical figures; add something like "about."

In the following sentence of your introduction in paragraph 2, there is no space between "and" and "accident." Review this quoted sentence:

"Various internal and external factors can affect driving, potentially leading to aggressive driving, errors, and accidents."

Please check all grammatical issues from the introduction to the end, as this is vital for your and the journal's reputation.

In the quoted sentence towards the end of your introduction, please remove the phrase "compared to conventional statistical techniques like regressions." The revised sentence should read:

"However, many ML models function as 'black box' systems, lacking interpretability, which limits their application in policymaking [16]."

Authors:

The authors sincerely thank you for your time, thoughtful review of our manuscript, and constructive feedback. We have thoroughly revised the manuscript, with particular attention to the grammatical issues you highlighted. We believe that the revised version now meets the standards of scientific writing and is suitable for publication. All changes have been clearly marked using track changes.

Editor:

When referencing your own tables and figures, please bold "Figure 1," "Table 1," and others. For example, when you mention it is further presented in Figure 1, it should be bolded as "Figure 1."

Authors:

Thank you for your feedback. We have bolded the names of figures and tables throughout the manuscript where they are referenced. We have ensured full alignment with the PLOS ONE submission guidelines (as outlined in https://journals.plos.org/plosone/s/file?id=9cba/PLOS%20Manuscript%20Body%20Formatting%20Guidelines.pdf). Specifically, we have referred to tables and figures as Table 1, Fig 1, S1 Table, S1 Fig, etc., in accordance with to PLOS ONE guideline.

Editor:

In your Statistical Analyses section, the first sentence states:

"Quantitative variables were described as the mean and standard deviation (SD), while qualitative variables were presented in the form of frequencies and percentages."

My question here is: how can qualitative variables be presented with percentages if they are not quantitative?

Authors:

Thank you for your attention to detail. The percentages for qualitative (categorical) variables were calculated by dividing the number of cases in a specific category by the total number of cases for that variable. For example, in Table 2, the percentage of error occurrences in accident hotspots under clear weather conditions is 1.86%. This was calculated by dividing the number of errors in accident hotspots under clear weather (3,570) by the total number of errors under clear weather conditions (191,570).

Editor:

I would be very glad if you could incorporate "hyperparameter optimization techniques" and show the ROC curve/AUC before and after tuning or optimization. You can employ only one technique, for example, grid search tuning or Bayesian optimization.

Authors:

Thank you for your insightful suggestion. We have performed grid search for hyperparameter tuning in last version of submission. For this version, We used the citation https://link.springer.com/article/10.1186/s12889-024-19566-8 to show the reason we choosed this approach in the "Machine learning models and evaluation" subsection of the method by:

"While various approaches such as grid search, random search, and Bayesian optimization exist for model optimization, grid search has demonstrated comparable performance. Therefore, it was selected for hyperparameter tuning in this study. Grid search was conducted using stratified 5-fold cross-validation with 3 repeats."

Although we had previously presented the results of hyperparameter tuning in S4 Table, we have now added the complete results for hyperparameter tuning of all six models in a new supplementary file (S5 Table).

Editor:

I recommend adding a strengths and limitations of the study section at the end of the discussion and before the conclusion.

Authors:

Thank you for your recommendation. It is added.

Editor:

Please also highlight headings and subheadings clearly, ensuring the first letter of each heading and subheading is capitalized.

Captions for each figure are essential.

Authors:

Thank you for your recommendation. We reviewed all headings and subheadings and have applied the appropriate formatting in accordance with the PLOS ONE guidelines. Additionally, figure captions have been provided in the manuscript following the journal's instructions, which recommend including figure legends in the text after their first mention.

Editor:

Lastly, the author may benefit from citing the following article for some of their methodological arguments, as it clearly articulates the aspects we should follow in machine learning, especially in predictive modeling; article link: https://link.springer.com/article/10.1186/s12889-024-19566-8

Authors:

Thank you for your recommendation. The referenced study is indeed valuable, particularly due to its comprehensive and detailed methodology. We have cited this reference as number 45 in our manuscript, in the section where we discuss the rationale for selecting grid search for model optimization.
